# Genetic Interruption of PD-1/PD-L1 as an Alternative Means for Immune Checkpoint Blockade in Cancer: A Review

**DOI:** 10.3390/pharmaceutics18060752

**Published:** 2026-06-18

**Authors:** Dan Li, Jiao Lu, Qianru Li, Huan Deng, Songwei Tan

**Affiliations:** 1 Hubei Provincial Corps Hospital of Chinese People’s Armed Police Force, Wuhan 430061, China; leedan12@126.com; 2 School of Pharmacy, Tongji Medical College, Huazhong University of Science and Technology, Wuhan 430030, China; m202576030@hust.edu.cn (J.L.); m202375837@hust.edu.cn (Q.L.); 3 School of Chemistry, Chemical Engineering and Life Sciences, Wuhan University of Technology, Wuhan 430070, China

**Keywords:** PD-1/PD-L1, gene therapy, tumor microenvironment, delivery systems

## Abstract

**Background/Objectives**: Immune checkpoints are critical regulatory pathways that maintain peripheral tolerance and prevent autoimmunity. Among these, the programmed death-1/programmed death-ligand 1 (PD-1/PD-L1) axis serves as a major inhibitory pathway that terminates T cell responses. While protein-based checkpoint blockade (ICB) targeting this axis has revolutionized clinical cancer therapy, its clinical efficacy is frequently limited by low response rates, immune-related adverse events (irAEs), and the emergence of adaptive resistance. To break through these bottlenecks, genetic interruption has emerged as a high-precision alternative to modulate the PD-1/PD-L1 pathway at the nucleotide level. **Methods**: A comprehensive systematic review of literature was performed across major databases (PubMed, Web of Science), with a focus on high quality studies published up to 2026. **Results**: Direct genomic disruption via CRISPR/Cas9 and post-transcriptional silencing through RNA interference can effectively neutralize inhibitory signaling at its source. Recent advances demonstrate that targeting upstream regulatory nodes—including metabolic checkpoints (e.g., lactate metabolism) and biophysical mechanisms (e.g., liquid–liquid phase separation)—provides superior transcriptional control over PD-L1. Furthermore, engineering CAR-T cells with multiplex gene editing (e.g., TCR/B2M/PD-1 knockout) or localized scFv secretion significantly enhances antitumor potency while reducing systemic toxicity. Innovations in organ-targeted lipid nanoparticles and stimuli-responsive biomimetic carriers further address the delivery barriers in solid tumors. **Conclusions**: Gene therapy provides a high-precision platform for PD-1/PD-L1 modulation, offering a viable strategy to overcome adaptive resistance. Future clinical application depends on the refinement of safer editing tools, such as base editing, and the standardization of intelligent delivery systems to ensure controllable and scalable cancer immunotherapy.

## 1. Introduction

Programmed death-ligand 1 (PD-L1, also known as B7-H1 or CD274) is a crucial inhibitory immune checkpoint protein in the immune system [1]. It binds to programmed cell death protein 1 (PD-1) to maintain the balance of immune cell functions. Under physiological conditions, the PD-1/PD-L1 pathway negatively regulates the immune system to prevent T cell overactivation and autoimmune diseases. However, cancer cells exploit this mechanism to escape immune surveillance through PD-L1 overexpression [2]. Within the tumor microenvironment (TME), PD-L1 is expressed by tumor cells as well as endothelial, epithelial, and myeloid cells, whereas PD-1 is highly expressed on tumor-infiltrating T cells, including exhausted T cells [3]. The binding of PD-L1 on cancer cells to PD-1 on T cells transmits inhibitory signals to effector T cells, inducing T cell exhaustion, while simultaneously providing anti-apoptotic signals to the tumor cells themselves to promote their survival, thereby severely suppressing the anti-tumor immune response [4].

Therapeutic strategies targeting immune checkpoints have been extensively applied in clinical oncology, with the primary clinical modality being immune checkpoint blockade (ICB), prolonging the overall survival of numerous patients and revolutionizing the cancer treatment [5,6,7]. Following the advent of the first cytotoxic T-lymphocyte-associated antigen 4 (CTLA-4) inhibitor, PD-1/PD-L1 inhibitors have been approved, with indications now covering a wide range of malignancies [8]. Although anti-PD-1/PD-L1 antibody monotherapy or its combination with adjuvant therapies can achieve significant responses and long-term remission in a subset of patients, its application is still limited by low response rates in certain cancer types, a lack of predictive biomarkers, immune-related toxicities, and drug resistance. The overall objective response rate remains around 40% [9]. Clinically, these standard anti-PD-1/PD-L1 antibodies are predominantly administered via intravenous (IV) infusion. The dosing regimens are typically administered either as weight-based doses (e.g., 2–3 mg/kg) or as flat fixed doses (e.g., 200 mg or 400 mg) every 2–6 weeks, depending on the specific agent and indication [10,11]. Among patients receiving anti-PD-1/PD-L1 therapies, the incidence of any-grade immune-related adverse events (irAEs) can reach up to 30%, manifesting as fatigue, rash, diarrhea, thyroid dysfunction, and even cardiotoxicity(incidence of approximately 1%,mortality rates 30–50%, only reversible at early stage) [12,13,14,15]. These inherent systemic toxicities and the constraints of systemic delivery routes underscore the urgent need for more controllable and localized intervention strategies, such as gene therapy. Emerging evidence indicates that tumor-intrinsic PD-L1 also exerts non-immune, pro-tumorigenic functions that are inaccessible to antibody blockade [16,17]. For instance, intracellular PD-L1 has been shown to enhance the DNA damage response and stimulate protective autophagy, thereby promoting tumor cell survival and fitness under stress [17].

Gene editing technology opens a new avenue for the regulation of the PD-1/PD-L1 pathway, which holds promise for reducing immune-related toxicities and providing new strategies to overcome acquired resistance to ICB [18]. In recent years, utilizing gene editing technology, particularly the CRISPR system, to directly modulate the PD-1/PD-L1 pathway has demonstrated feasibility and potential for achieving efficient anti-tumor immunity [19,20]. On one hand, the knockout of PD-L1 or PD-1 genes in tumor or immune cells can block this inhibitory signal at the source and enhance the anti-tumor activity of T cells [21]. On the other hand, editing technology can be used to engineer immune cells (such as CAR-T cells) to achieve localized, specific immune enhancement while avoiding systemic immune over activation [22]. Furthermore, using gene editing to construct or screen preclinical models—such as syngeneic mouse models, genetically engineered mouse models (GEMMs), and patient-derived organoids (PDOs)—can help elucidate the molecular mechanisms of resistance to conventional immune checkpoint blockade (ICB) and therapy-associated toxicity, thereby guiding better combination therapy strategies [23,24]. However, the clinical translation of this technology still faces major challenges, such as editing efficiency, off-target effects, delivery systems, and long-term safety [25,26]. In the future, by developing novel delivery vectors and optimizing editing strategies, gene editing is expected to integrate deeply with existing immunotherapies, ultimately achieving more effective cancer immunotherapy.

**Figure 1:**
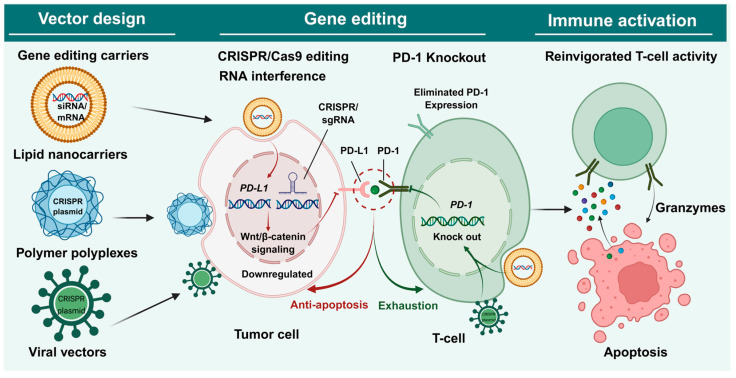
Schematic overview of the PD-1/PD-L1 immune checkpoint axis and the corresponding gene-editing therapeutic strategies. The binding of PD-L1 to PD-1 induces T cell exhaustion and facilitates tumor immune evasion. Various gene delivery vectors (e.g., lipid nanocarriers, polymer polyplexes, and viral vectors) are designed to deliver CRISPR/Cas9 systems or nucleic acids. By knocking out the PD-1 gene in T cells or the PD-L1 gene in tumor cells, the inhibitory signaling is abrogated, leading to robust T cell activity and subsequent tumor cell apoptosis.

In summary, immune checkpoint therapies centered on the PD-1/PD-L1 pathway have shown significant clinical efficacy. However, the prevalent issues of insufficient response rates, immune-related toxicities, and drug resistance limit their long-term application. To break through these bottlenecks, research focuses are gradually shifting toward more controllable intervention strategies for this pathway. This review will comprehensively explain the complex regulatory mechanisms of the PD-1/PD-L1 pathway, laying a theoretical foundation for subsequent discussions on gene editing-based precision treatment strategies. Meanwhile, we summarize emerging therapeutic strategies that regulate PD-1/PD-L1 expression and function at the genetic level, focusing on the latest advances in gene therapy approaches such as gene editing (e.g., CRISPR/Cas systems), gene silencing (e.g., RNA interference), and gene delivery vectors (Figure 1, Table 1). These approaches target PD-1/PD-L1 signaling in tumor or immune cells to enhance anti-tumor immune effects, reduce side effects, and overcome resistance. By reviewing the mechanisms of action, preclinical findings, and challenges of these strategies, this review aims to provide a theoretical basis and outlook for developing the next generation of cancer immunotherapies.

## 2. Mechanisms of the PD-1/PD-L1 Pathway in Tumors

In TME, the expression of PD-1 is tightly regulated at multiple molecular levels. Following T cell receptor (TCR) activation, PD-1 transcription is positively regulated via the calcium-NFATc1 pathway and TGF-β signaling. Pro-inflammatory cytokines, such as IL-6 and IL-12, enhance PD-1 transcription by activating STAT3/STAT4 [50]. Additionally, IL-7, IL-15, and IFN-α participate in this regulatory network through distinct signaling pathways. This sophisticated regulatory network serves as a protective mechanism to prevent immune overactivation under physiological conditions; however, it also becomes a critical factor in tumor immune escape. The complete molecular mechanisms underlying this process remain to be further elucidated [51,52].

The binding of PD-L1 expressed by cancer cells to PD-1 on the surface of T cells induces T cell inactivation or apoptosis, leading to tumor immune evasion [53]. The PD-1/PD-L1 axis is not merely a simple “on-off” switch but a dynamic process finely tuned by multiple signals. T cell exhaustion is frequently observed in cancer patients, with the PD-1/PD-L1 pathway being a key driver of this state. When PD-1 and the T cell receptor (TCR) bind to their respective ligands, activated TCR-proximal Src kinases phosphorylate the immunoreceptor tyrosine-based switch motif (ITSM) and the immunoreceptor tyrosine-based inhibitory motif (ITIM) of PD-1 [54,55]. Subsequently, SHP2 is recruited via the ITSM (primary) and ITIM (auxiliary) motifs, interacting with PD-1 in a bivalent form (SHP2-cSH2). Recent biochemical evidence suggests that the PD-1/SHP2 complex preferentially dephosphorylates the co-stimulatory receptor CD28 rather than the TCR itself, identifying CD28 as a primary target for PD-1-mediated immunosuppression [56]. This process inhibits the phosphorylation of downstream TCR signaling molecules, such as CD28 and ZAP70, thereby suppressing the PI3K/AKT signaling pathway [57]. Beyond inhibiting early T cell activation signals, PD-1 can directly impair the antigen recognition capability of T cells by reducing the stability of the TCR-pMHC-CD8 ternary complex [58].

**Figure 2:**
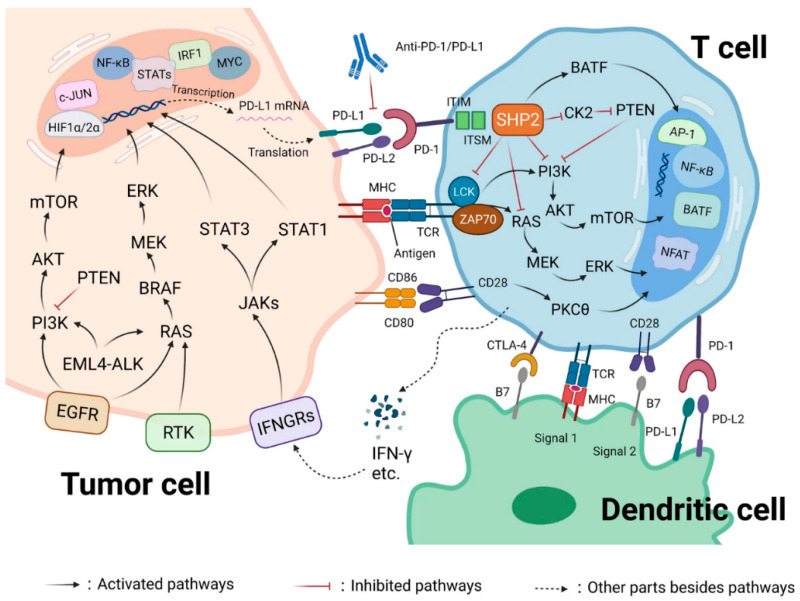
PD-1/PD-L1 interaction mediated T cell inhibition. The RAS/MEK/ERK, PI3K/Akt/mTOR, JAK/STATs signaling and TLRs/IKKs pathways are the main pathways regulating PD-L1 expression. IRF1, STATs, MYC, NF-κB, c-Jun and HIF1α/2α are the main downstream transcription factors. Posttranslational modifications of PD-L1 include phosphorylation, ubiquitination, glycosylation and palmitoylation. Induction of PD-L1 by cytokines, such as IFN-γ, is considered a secondary mechanism. Activation of PD-1/PD-L1 signaling leads to the recruitment of the phosphatase SHP-2 to the C-terminal of the ITSM, which downregulates the RAS-MEK-ERK and PI3K-Akt-mTOR pathways and attenuates LCK-induced phosphorylation of ZAP70. In addition, SHP-2 induces the expression of BATF, which inhibits the expression of some effector genes. In general, activation of PD-1/PD-L1 signaling leads to the inhibition of T cell proliferation and activation. Activation of PD-1/PD-L1 can be blocked by anti-PD-1/PD-L1 antibodies. In addition, APCs uptake tumor antigens and regulate T cell responses through the interaction between major MHC and TCRs. APCs (dendritic cells) regulate T cell activity through modulating the interaction between PD-L1/PD-L2 and PD-1 and the interaction between B7 and CD28. CTLA-4 is a negative regulator of costimulation that is activated in the recognition of specific tumor antigens presented by APCs. Reproduced with permission [2]. Copyright 2022 Springer Nature by CC-BY 4.0.

Current research indicates that PD-L1 expression in cancer cells is regulated by diverse mechanisms (Figure 2). Its aberrant overexpression can be driven by genomic alterations as well as the constitutive activation of oncogenic pathways, including RAS-MEK-ERK, PI3K-Akt-mTOR, JAK-STAT, and TLR-IKK. In addition to regulation by intrinsic mutations, PD-L1 can be synergistically upregulated by TME cytokines such as IFN-γ, TNF-α, and TGF-β [1,59]. It can also be triggered by the loss or inactivation of tumor suppressor genes, such as PTEN and RB [60,61]. Furthermore, abnormal elevations in PD-L1 protein levels may stem from dysregulation in its proteolytic degradation processes [48,49]. In addition, the stability of PD-L1 is heavily dependent on post-translational modifications and specific regulatory proteins. N-linked glycosylation has been shown to stabilize PD-L1 by preventing its 26S proteasome-mediated degradation, thereby promoting tumor immune evasion [62]. In parallel, the transmembrane protein CMTM6 (and its paralog CMTM4) has been identified as a key chaperone that co-localizes with PD-L1 in recycling endosomes, effectively shielding PD-L1 from lysosomal degradation and ensuring its high-level presence on the plasma membrane [48].

While PD-L1 is abnormally overexpressed in various tumors, its expression levels vary significantly across different tumor types. For instance, immunologically “hot” tumors (e.g., melanoma and non-small-cell lung cancer) typically exhibit robust PD-L1 expression, making direct interruption of PD-1/PD-L1 highly effective. For “cold” malignancies, such as pancreatic cancer, often display highly heterogeneous or restricted expression profiles. This profound variability is principally governed by two dimensions: intrinsic oncogenic drivers (e.g., PTEN loss or aberrant RAS/MEK signaling) and extrinsic TME factors (e.g., adaptive induction by IFN-γ secreted by infiltrating immune cells or tissue hypoxia) [63,64,65,66]. For these “cold” tumors, direct PD-1/PD-L1 mono-targeting is insufficient. Multi-modal strategies combining upstream pathway disruption with immunogenic cell death (ICD) are required to convert “cold” tumors into “hot” ones. Despite this heterogeneity, when aberrantly present, it serves as a pivotal immune evasion mechanism by suppressing anti-tumor immune responses via the PD-1/PD-L1 pathway, which is closely associated with poor clinical prognosis [67]. Rather than merely serving as a theoretical model, ICB strategies targeting this pathway—specifically anti-PD-1/PD-L1 monoclonal antibodies (e.g., pembrolizumab and nivolumab)—have translated into unprecedented clinical milestones. For instance, in advanced melanoma, PD-1 blockade has dramatically shifted the 5-year overall survival rate from historically less than 10% to over 50%. Similarly, these therapies have become the standard first-line treatment for advanced non-small-cell lung cancer (NSCLC), offering durable remission and significantly extending median overall survival compared to traditional chemotherapy [68,69,70]. However, this strategy has inherent limitations. First, antibody drugs only block the ligand-receptor interaction and cannot modulate the sustained intracellular inhibitory signaling pathways [71]. Second, treatment may trigger widespread immune activation, leading to immune-related adverse events (irAEs) [72]. Finally, primary and acquired resistance issues are prominent, and the overall objective response rate remains limited [73]. Additionally, while PD-L1 expression in the TME can partially predict response to ICB, clinical responses do not always align with expression levels: some PD-L1-positive patients do not respond, whereas some PD-L1-negative patients may still benefit [66].

Beyond its well-established role on the plasma membrane, recent evidence has revealed that PD-L1 undergoes dynamic nucleocytoplasmic translocation, which serves as a critical mechanism for immune evasion and therapy resistance. This process is orchestrated by p300-mediated acetylation and HDAC2-dependent deacetylation, which facilitate the interaction of PD-L1 with endocytosis and nucleocytoplasmic transport machinery. Once in the nucleus, PD-L1 functions as a transcriptional co-regulator, and its deficiency leads to the compromised expression of multiple immune-response-related genes. Notably, blocking this nuclear translocation has been shown to reprogram the immune landscape and synergistically enhance the efficacy of PD-1 blockade [74]. Since conventional monoclonal antibodies are inherently limited to sequestering membrane-bound PD-L1, they remain ineffective against these intrinsic nuclear functions.

These limitations suggest that targeting the PD-1/PD-L1 protein alone may be insufficient to overcome tumor heterogeneity and adaptive resistance. Therefore, direct intervention at key nodes of its upstream regulatory network is a more fundamental and controllable strategy, that is, gene therapy strategies such as CRISPR-mediated knockout or RNAi-based silencing to deplete the total cellular pool of PD-1/PD-L1, thereby neutralizing both its extrinsic immunosuppressive signaling and its intrinsic nuclear-mediated transcriptional activities. In this way, one can knockdown or modify specific genes driving aberrant PD-L1 expression (such as oncogenic signaling components or epigenetic regulators) or engineer T cells to modulate PD-1 expression. This strategy holds the potential for spatiotemporally specific precision intervention. Indeed, precedent studies utilizing advanced targeted delivery platforms—such as stimuli-responsive polymeric nanocarriers or self-assembling peptide systems—have delivered gene-editing tools or RNAi machinery to challenging orthotopic or metastatic tumor models (e.g., orthotopic pancreatic or breast tumors). These sophisticated platforms have demonstrated robust intratumoral immune activation and significant tumor regression, while exhaustive in vivo biosafety evaluations (including blood biochemistry and histological analyses) revealed negligible off-target systemic toxicities [75,76]. Consequently, such spatiotemporal intervention expands the therapeutic window by maximizing antitumor efficacy while minimizing side effects, offering new avenues to overcome drug resistance.

## 3. Tumor Gene Therapy Strategies Based on PD-1/PD-L1 Blockade

### 3.1. Targeting PD-1 Receptors on Immune Cells

Interventions targeting PD-1 receptors on immune cells primarily focus on downregulating PD-1 expression to rejuvenate T cell functions through advanced delivery systems or cellular engineering. Based on their mechanisms and delivery modalities, these strategies are categorized into the following three classes.

First, self-blocking strategies are mediated by emphasizing the localized expression of PD-1 on tumor cells to competitively sequester PD-L1 signals. Pan et al. developed a bacterial outer membrane vesicle (OMV) system modified with LyP1 peptides (LOMV) to encapsulate PD-1 plasmids [77]. This nanocarrier specifically accumulates in tumor tissues following intravenous injection and induces tumor cells to express PD-1 protein. The surface-expressed PD-1 subsequently binds to PD-L1 on the same or adjacent tumor cells, effectively blocking inhibitory signals and reactivating cytotoxic T lymphocytes (CTLs). Furthermore, the OMV components act as adjuvants to recruit immune cells and promote IFN-γ secretion, facilitating the differentiation of T cells into central memory T cells to establish long-term immune memory.

**Figure 3:**
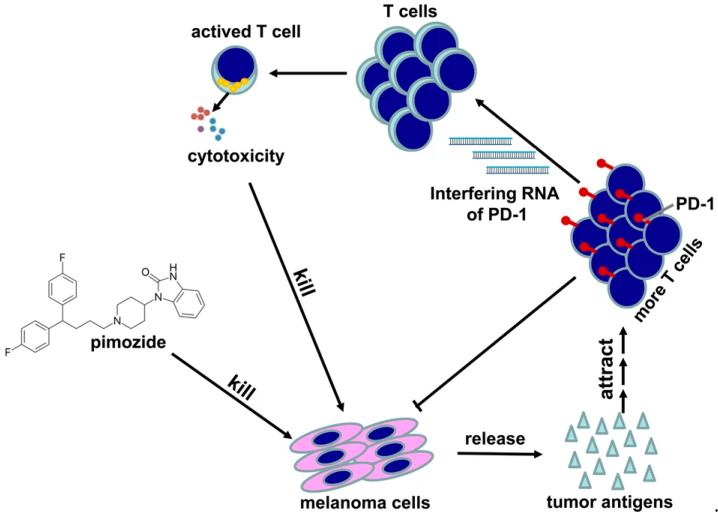
Schematic overview of the synergistic antimelanoma mechanisms of combination therapy with pimozide and pSi-PD-1. By blocking the PD-1 pathway via RNA interference, the therapy restores the cytotoxic killing function of the activated T cells. The combined actions of direct tumor cell killing by pimozide and the enhanced T cell-mediated cytotoxicity via PD-1 inhibition play a synergistic antitumor role in eradicating melanoma cells. Reproduced with permission [78]. Copyright 2019 Springer Nature by CC-BY 4.0.

Second, RNA interference (RNAi)-based gene silencing strategies. Zhao et al. utilized attenuated Salmonella to deliver PD-1 siRNA, demonstrating that silencing PD-1 effectively activates anti-tumor immunity and inhibits tumor growth in melanoma models (Figure 3) [78]. To further enhance therapeutic performance, Gao et al. constructed a gadolinium (III)-chelated gold dendrimer (Gd-Au DENP-PS) with excellent stability and biocompatibility [79]. This platform achieved high siRNA loading and enhanced serum stability, enabling efficient PD-1 silencing in T cells within both melanoma and aged mouse models. This nanocomplex reversed T cell exhaustion and significantly increased effector CD8^+^ and CD4^+^ T cell populations, providing a novel platform for CT/MR dual-mode imaging-guided immunotherapy.

However, despite these systemic interventions, unselective PD-1 silencing in in vivo T cell populations presents formidable clinical drawbacks that must be rigorously addressed. First, PD-1 is a fundamental safeguard for peripheral tolerance; removing this critical brake on auto-reactive T cells globally inevitably precipitates severe, widespread autoimmune toxicities (irAEs) in healthy tissues. Second, unlike protein-based blocking antibodies with a finite biological half-life, genetic silencing—particularly through permanent genome editing tools like CRISPR/Cas9—induces irreversible alterations, making subsequent autoimmune complications exceptionally difficult to manage clinically. Third, the permanent absence of PD-1 signaling can paradoxically undermine long-term efficacy; persistent and unregulated T cell receptor (TCR) hyperactivation often drives T cells toward premature exhaustion or activation-induced cell death (AICD), ultimately depleting the immunological memory pool.

Collectively, these severe drawbacks underscore the urgent necessity of developing highly targeted, microenvironment-responsive delivery vectors. Emerging platforms leveraging advanced high molecular materials, such as dynamically reconfigurable peptide self-assembly systems or in situ biomineralization strategies, offer compelling solutions. These intelligent carriers can physically shield the gene-editing payload during systemic circulation and undergo triggered release strictly within the desmoplastic matrix of “cold” malignancies, such as pancreatic cancer. Validating the spatiotemporal precision and systemic safety of these tailored interventions in fully immunocompetent syngeneic settings—for instance, utilizing Panc02 cell line-derived models—will be critical for future clinical translation.

Driven by this imperative to balance potent efficacy with rigorous safety, third, composite functional strategies integrate PD-1 modulation with immune cell engineering to enhance therapeutic efficacy and safety. Hamilton et al. generated CAR-T cells by co-delivering CAR mRNA and PD-1-targeted siRNA using ionizable lipid nanoparticles (LNPs) [80]. This non-viral approach allows for transient CAR expression and temporary PD-1 blockade, thereby reducing the risk of long-term off-target effects and avoiding the irreversible toxicity associated with permanent PD-1 ablation. Furthermore, to address the formidable physical barriers that often impede these engineered cells, Ding et al. combined the tumor-penetrating peptide iRGD with PD-1-knockout adoptive lymphocyte therapy (PD1-KO-CTL) [81]. By improving T cell infiltration into tumor tissues. This strategy offers a potential solution to the infiltration bottleneck commonly encountered in the treatment of solid tumors.

### 3.2. Strategies Targeting the PD-L1 on Tumor Cells

With the rapid evolution of gene-editing technologies, therapeutic strategies targeting PD-L1 on cancer cells have advanced from simple gene knockout to multi-functional, precision systems. These modern platforms integrate stimuli-responsive activation, immune microenvironment remodeling, and intelligent delivery technologies.

#### 3.2.1. Light-Responsive CRISPR Delivery Systems

Spatiotemporal control of gene-editing activity is essential to minimize off-target effects. Zhao et al. developed a gene-editing system (F-PC/pHCP) comprising a fluorinated dendrimer carrier loaded with chlorin e6 (Ce6) and a CRISPR/Cas9 plasmid driven by the HSP70 promoter [27]. Upon 660 nm laser irradiation, the generated reactive oxygen species (ROS) trigger the HSP70 promoter to drive Cas9 expression, leading to the permanent disruption of the PD-L1 gene, which was validated both in vitro in cancer cells and in vivo in tumor-bearing mice. Similarly, another light-controlled system utilized a photo-activatable self-degradable polyethylenimine derivative (PPCe) and a CRISPR/Cas9 plasmid (pX330/sgPD-L1) [28]. Under 660 nm illumination, this system facilitates endosomal escape via photochemical internalization and releases the plasmid through PPCe photodegradation, achieving PD-L1 editing in both cancer cells and cancer stem cells under both in vitro and in vivo settings. Furthermore, Tang et al. employed a NIR-II light-triggered thermo-responsive strategy [29]. By delivering a heat-inducible CRISPR/Cas9 system via cationic gold nanorods, they achieved specific PD-L1 knockout in deep-tissue in vivo mouse models under mild hyperthermia while simultaneously inducing ICD to reprogram the tumor microenvironment and promote T cell infiltration, thereby enhancing the efficacy of anti-PD-1/PD-L1 immunotherapy.

Despite these preclinical results, a major bottleneck for the clinical translation of light-responsive platforms is the limited tissue penetration depth of conventional light sources, particularly in less accessible or deep-seated tumors. To overcome this hurdle, several cutting-edge strategies are being actively explored. First, transitioning from visible light to the second near-infrared window (NIR-II, 1000–1700 nm) significantly minimizes photon scattering and tissue absorption, extending the effective penetration depth to several centimeters into solid tissues [29]. Second, from a clinical perspective, light-responsive systems can be coupled with existing interventional medical devices, such as fiber-optic endoscopes, laparoscopes, or interstitial optical fiber needles. These devices allow for the minimally invasive, direct delivery of laser energy to deep anatomical sites, such as pancreatic, colorectal, or hepatic tumors. Lastly, the incorporation of upconversion nanoparticles (UCNPs) within the nanocarriers offers an elegant biophysical solution; UCNPs can absorb deeply penetrating low-energy NIR light and locally convert it into high-energy visible or UV light to trigger the photo-responsive gene-editing vectors in situ. Collectively, these advancements hold the potential to expand the utility of light-triggered CRISPR delivery from superficial lesions to complex, deep-seated malignancies.

#### 3.2.2. Tumor Microenvironment-Responsive Strategies

To enhance tumor specificity and minimize off-target effects in normal tissues, “intelligent” nanocarriers have been engineered to respond to the unique physicochemical signatures of the TME, such as acidity (pHe), hypoxia, and elevated redox levels. Zhang et al. proposed a “dual-locked” nanoparticle (DLNP) that integrates pH and H_2_O_2_ responsiveness [30]. The platform only unlocks and releases its CRISPR/Cas13a payload upon the simultaneous detection of acidic pHe and high H_2_O_2_ concentrations, significantly improving internalization efficiency and preventing off-target activation in normal tissues. Beyond acidity, hypoxia is a hallmark of the TME that frequently drives PD-L1 upregulation via HIF-1α signaling. Qu et al. developed a light-enhanced, hypoxia-responsive nanoplatform for the co-delivery of CRISPR/Cas9 targeting both HIF-1α and PD-L1. By utilizing hypoxia-sensitive azobenzene bridges, the system achieves controllable cargo release within the oxygen-depleted tumor core, effectively reversing hypoxia-induced immune resistance and synergizing with photodynamic therapy [31]. To address the formidable biological barriers in complex cancers like glioblastoma (GBM), cascade-responsive strategies have been employed. Deng et al. designed a cascade-responsive nanoparticle (GCNP) that reacts to the sequential triggers of tumor acidity and high intracellular glutathione (GSH). This multi-stage activation ensures that the CRISPR/Cas9 machinery is specifically deployed after penetrating the blood–brain barrier and entering GBM cells, enabling high-precision PD-L1 knockout with minimal systemic toxicity [32].

#### 3.2.3. Synergistic Multimodal Therapy

**Figure 4:**
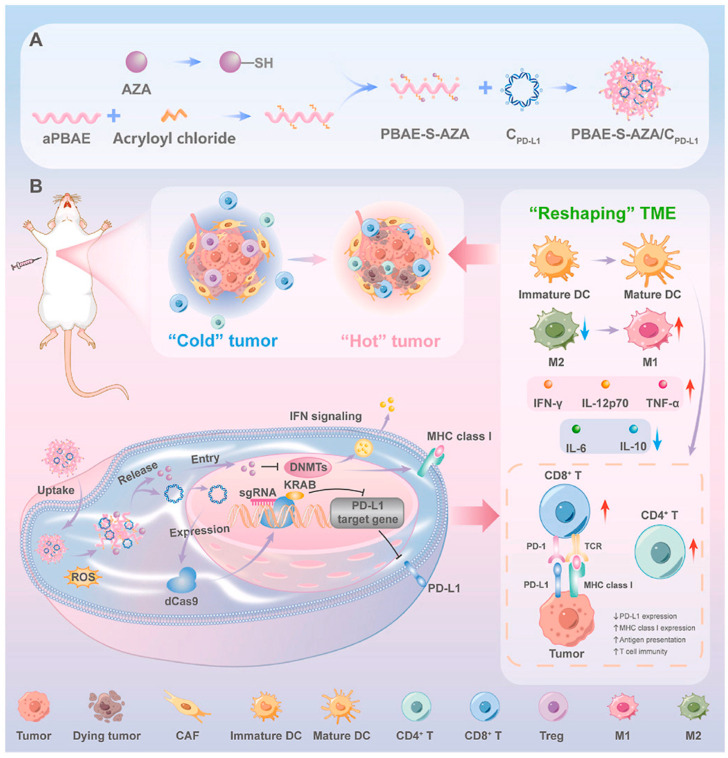
Schematic illustration of the ROS-responsive prodrug polymer complex (PBAE-S-AZA/CPD-L1) co-delivering a CRISPRi system and an epigenetic inhibitor for remodeling the tumor immune microenvironment. (**A**) Construction of the ROS-responsive polyplex nanoparticles and co-delivery of AZA and PD-L1-targeting CRISPRi plasmid (CPD-L1). (**B**) Mechanisms of complex-mediated PD-L1 downregulation and TME remodeling: the high intracellular ROS levels trigger the oxidation and rapid hydrolysis of the thioether bonds on the polymer side chains, leading to the synchronous and responsive release of the AZA drug molecules and CRISPRi plasmids. AZA inhibits DNA methyltransferases (DNMTs), upregulates the expression of MHC class I molecules, enhances tumor antigen presentation, and promotes the maturation of immature dendritic cells (Immature DCs) into mature DCs (Mature DCs). The synergistic effect of gene editing and epigenetic regulation significantly activates the antitumor immunity of CD8^+^ and CD4^+^ T cells, promotes the secretion of inflammatory cytokines (e.g., IFN-γ), downregulates Tregs, and drives macrophage polarization from the M2 to the M1 phenotype. Ultimately, the immunosuppressive “cold tumor” is remodeled into an immunologically active “hot tumor” with robust tumoricidal efficacy. Reproduced with permission [84]. Copyright 2025 Elsevier by CC-BY 4.0.

Combining PD-L1 knockout with innate immune activation can reshape the immunosuppressive TME. Lu et al. developed a hyaluronic acid-modified hollow manganese dioxide platform (HMnMPH) to co-deliver a STING agonist (MSA-2) and CRISPR/Cas9 plasmids [82]. In the acidic, high-GSH TME, the HMnMPH degrades to release Mn^2+^ and MSA-2, which synergistically activate the cGAS-STING pathway. This, combined with PD- L1 knockout, reverses T cell exhaustion and induces long-term immune memory. Additionally, Zhang et al. utilized a biomimetic platform (UR@M) based on ursolic acid (UA) to deliver the CRISPR/Cas9 system [83]. This platform leverages UA to activate the TLR2-MyD88-TRAF6 pathway, achieving a triple effect: tumor targeting, gene editing, and innate-adaptive immune synergy. Similarly, we designed ROS-responsive prodrug polyplexes to co-deliver the CRISPRi system and azacytidine (AZA, an epigenetic inhibitor) (Figure 4) [84]. This platform achieved transcriptional silencing of PD-L1 while simultaneously remodeling the “cold” tumor microenvironment of triple-negative breast cancer. The ROS-triggered drug release ensured that gene interference and epigenetic modulation occurred synergistically within the tumor site, maximizing therapeutic efficacy.

#### 3.2.4. Ribonucleoprotein (RNP) Delivery Strategies

Direct delivery of Cas9 RNP complexes (protein + sgRNA) offers a distinct clinical translation advantage compared to plasmid-based delivery, which often leads to the prolonged intracellular persistence and expression of Cas9. The transient presence of the Cas9 protein ensures that the gene-editing machinery is degraded shortly after performing its function, thereby significantly narrowing the temporal window for off-target cleavage and mitigating long-term genomic safety concerns. For instance, Fang et al. constructed pH responsive calcium phosphate nanoparticles (CaP NPs) for delivering Cas9/sgRNA ribonucleoproteins (RNPs) targeting PD-L1 and Gal-9 genes [85]. This PG–RNP@CaP NPs are delivered through intratumoral injection, responsive to the release of RNPs within tumor cells, achieving efficient knockout of PD-L1 and Gal-9, thereby enhancing T cell activity. Meanwhile, the degradation of CaP NPs leads to the accumulation of intracellular Ca^2+^, triggering the release of damage related molecular pattern signals, activating natural immune cells such as dendritic cells, and enhancing antigen presentation. This local genome editing strategy significantly reduces the risk of off target editing throughout the body while activating local and systemic T cell anti-tumor effects. Lu et al. designed a system using mesoporous polydopamine (mPDA) coated with Fe_3_O_4_ nanoparticles for magnetic-targeted RNP delivery [86]. This system enhances tumor enrichment through magnetic targeting and utilizes photothermal effects to induce immunogenic cell death, release Cas9 RNP to knock down PD-L1, thereby activating anti-tumor immunity. Ji et al. developed a light-triggered singlet oxygen-responsive nanoplatform for the targeted knockout of PD-L1 in triple-negative breast cancer (TNBC). This biomimetic system, coated with tumor cell membranes, utilizes a singlet oxygen-cleavable linker to tether Cas9 RNPs. Upon second near-infrared (NIR-II) irradiation, the generated singlet oxygen triggers the immediate release of the RNP for PD-L1 disruption while simultaneously inducing photodynamic therapy (PDT). This dual action effectively reverses the immunosuppressive TME and amplifies the antitumor immune response [87]. Moreover, organ-specific RNP delivery is still a challenge. To Addressing this, A major bottleneck for in vivo CRISPR applications is the natural sequestration of delivery vehicles in the liver. To address this, Haley et al. employed high-throughput molecular barcoding alongside traditional screening to identify lipid nanoparticle (LNP) formulations specifically optimized for RNP delivery. This approach yielded a highly potent lung-tropic LNP capable of efficient gene editing within pulmonary endothelial and epithelial cells. Notably, the formulation exhibited high specificity for the lung with zero off-target indel formation in the liver, representing a significant milestone in overcoming delivery barriers for extrahepatic and clinically relevant genomic targets [88].

#### 3.2.5. RNA Interference (RNAi)-Based Silencing

RNAi remains a cornerstone for post-transcriptional PD-L1 silencing, which is thought to be more convenient and easier to design than antibody drugs [89]. To overcome the instability of naked siRNA and enhance intracellular bioavailability, encapsulating them in certain delivery systems is a common and effective strategy, and various functionalized systems have been developed [90].

1. Lipid-Based and Lipid-Polymer Hybrid Carriers

Lipid-based and lipid-polymer hybrid carriers are widely utilized due to their excellent biocompatibility and versatile functionalization. Wang et al. developed a polymer-lipid hybrid nanovesicle (P/LNVs) platform that enhances endogenous tumor antigen presentation through doxorubicin-induced immunogenic chemotherapy [33]. Simultaneously, the platform utilizes siPD-L1 to downregulate PD-L1 expression in cancer cells, significantly increasing CD8^+^ T cell infiltration and remodeling the immunosuppressive microenvironment. This system exhibited superior anti-tumor efficacy across prevention, metastasis, and solid tumor models. Similarly, Lian et al. addressed the co-overexpression of CD47 and PD-L1 in tumor cells by designing an EpCAM-targeting cationic liposome (LPP-P4-Ep) [34]. By simultaneously knocking down CD47 and PD-L1 proteins via RNAi, this carrier blocks the dual CD47/SIRP-α and PD-L1/PD-1 inhibitory pathways. The study validated its anti-tumor efficacy in PC-9 lung cancer cells, 4T1 mouse models, and lung metastasis models, while confirming safety through systemic toxicity evaluations after multiple administrations. To overcome the limitations of incomplete checkpoint inhibition, Hu et al. developed a PD-1-functionalized hybrid vesicle-liposome platform (PD-1-HVL-siKAT8) [35]. This biomimetic system delivers KAT8 siRNA to disrupt liquid–liquid phase separation (LLPS) [35], and thus achieves PD-L1 blockade and promotes M1 macrophage polarization. Moreover, ^10^B-containing polymer-lipid hybrid nanoparticles was designed for PD-L1 siRNA delivery in the context of Boron Neutron Capture Therapy (BNCT) to achieve the synergy between radiotherapy and gene silencing [36]. Unlike traditional antibodies, the delivered siRNA suppressed the compensatory upregulation of intracellular PD-L1 induced by neutron irradiation. Crucially, the silencing of PD-L1 also inhibited DNA damage repair, thereby sensitizing tumor cells to BNCT and amplifying the subsequent ICD to reshape the cold TME.

2. Inorganic Nanomaterial Carriers

**Figure 5:**
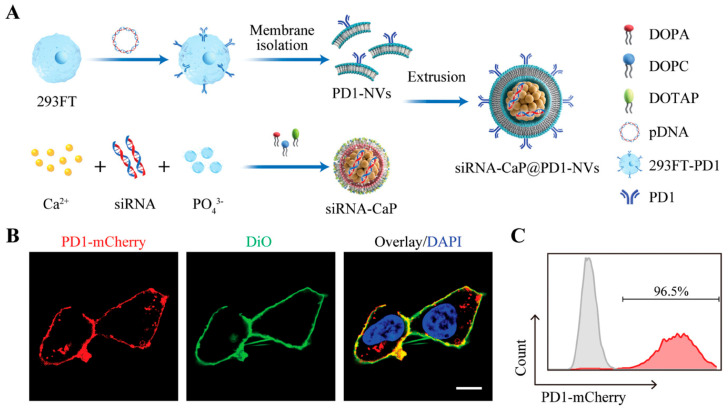
Schematic illustration of multifunctional biomimetic nanocarriers for dual-targeted immuno-gene therapy. (**A**) Schematic illustration of the synthesis process of siRNA-CaP@PD1-NVs. (**B**) Confocal images of HEK293FT cells stably expressing fusion protein PD1-mCherry (red) on cell membrane, cell membrane was stained by DiO (green). Scale bar:10 µm. (**C**) The representative FCM profiles of PD1-expressing HEK293FT stable cells (gated by mCherry+). Reproduced with permission [39]. Copyright 2024 Wiley by CC-BY.

Inorganic nanocarriers, such as graphene oxide, calcium phosphate, and Prussian blue, have garnered attention for their structural stability, ease of functionalization, and unique biological effects. Li et al. utilized graphene oxide (GO) to construct GO-PEI-PEG/siPD-L1 complexes [37]. Combined with sorafenib, this material remodeled the tumor immune microenvironment, promoted CD8^+^ T cell infiltration, and induced tumor ferroptosis, thereby synergistically inhibiting hepatocellular carcinoma (HCC) progression and enhancing the anti-tumor immune response. Tang et al. developed a hollow Prussian blue (HPB) nanocarrier (HPB-S-PP@LOx/siPD-L1) for the co-delivery of lactate oxidase (LOx) and siPD-L1 [38]. Upon accumulation in the tumor via the EPR effect, the carrier responsively released its payload in the high-glutathione (GSH) environment. Facilitated by the carrier’s intrinsic catalytic oxygen production, LOx efficiently consumed lactate in the TME to alleviate acidosis and reverse immunosuppression, while siPD-L1 enhanced immune checkpoint blockade in 4T1 triple-negative breast cancer models. Sun et al. developed a virus-like multifunctional nanocarrier (siRNA-CaP@PD1-NVs) consisting of a PD-L1/Pbrm1 siRNA core, a calcium phosphate (CaP) capsid, and a PD1-modified membrane envelope (Figure 5) [39]. Experiments confirmed the expression of PD1 protein on HEK 293FT cells. The carrier facilitated endosomal escape and tumor-targeted siRNA delivery, while simultaneously activating dendritic cells via calcium ion adjuvants and blocking the PD-L1 pathway through PD1 decoys. Similarly, Huang et al. constructed tumor-targeted lipid-dendrimer-calcium phosphate nanoparticles (TT-LDCP) that serve both as efficient gene delivery vehicles and immune adjuvants [40]. The thymine-functionalized dendrimers on the surface synergistically activated the STING-cGAS pathway. This platform co-delivered PD-L1 siRNA and IL-2 plasmids to HCC cells, achieving nanotechnology-driven dual targeting to selectively reprogram the immunosuppressive microenvironment and improve immunotherapy efficacy.

3. Stimuli-Responsive Delivery Systems

Stimuli-responsive systems integrate components sensitive to pH, ROS, or other triggers to achieve specific release within the tumor microenvironment. Guan et al. loaded plasmid DNA expressing PD-L1 small hairpin RNA (shPD-L1) into pH-responsive, dual charge/size-reversal P[(GP)D] nanoparticles, achieving effective silencing of PD-L1 in B16F10 cells [41]. Li et al. developed a tri-block polymer, PDDT (comprising PT, pH-responsive P(DPA-co-DMAEMA), and PEG), whose pH-responsive hydrophobic core can be tuned via the DPA/DMAEMA ratio [42]. This design significantly promoted endosomal escape and cytosolic release of siRNA, effectively restoring immune surveillance in CT-26 tumor models. Wan et al. developed T7 peptide-modified, ROS-responsive nanoparticles for the co-delivery of siPD-L1 and doxorubicin (Dox) [43]. By targeting the transferrin receptor overexpressed on tumor cells, the carrier enhanced cellular uptake and triggered rapid Dox release in the high-ROS intracellular environment. Simultaneously, siPD-L1 blocked PD-1/PD-L1 signaling and promoted T cell proliferation, demonstrating superior anti-tumor effects compared to Dox-only nanoparticles in 4T1 models. Finally, Zhang et al. constructed multifunctional redox-responsive nanoparticles (M+C+siPD-L1) loaded with a TLR agonist (M), catalase (C), and siPD-L1. These nanoparticles synergistically achieved the repolarization of tumor-associated macrophages (TAMs) toward the M1 phenotype, the clearance of intratumoral ROS, and the silencing of PD-L1, effectively reversing Treg-mediated suppression of CD8^+^ T cell function [44].

### 3.3. Regulation of Upstream Signaling Pathways

Beyond the direct targeting of PD-L1 mRNA or genomic sequences, intervening in the upstream signaling pathways that govern PD-L1 expression, transcription, and post-translational stability has emerged as a more sophisticated strategy for remodeling the TME.

**Figure 6:**
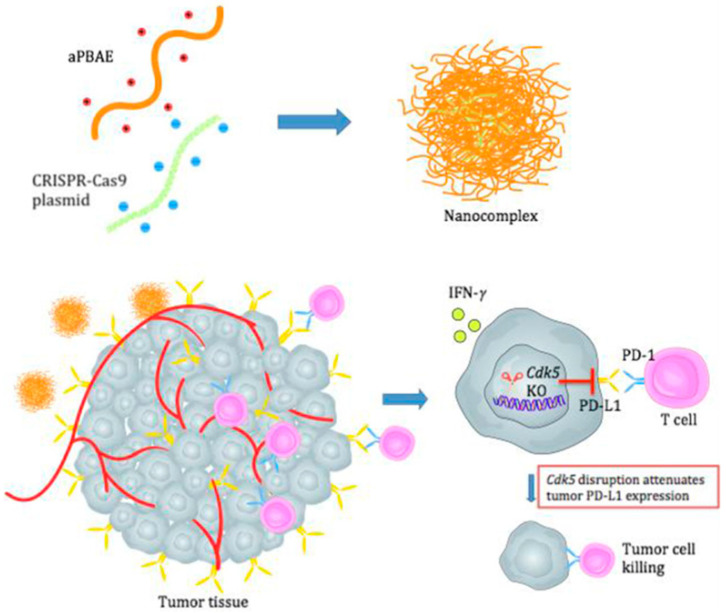
Schematic illustration of aPBAE/CRISPR-Cas9 nanocomplex preparation and the mechanism of PD-L1 attenuation and enhanced anti-tumor immunity. The CRISPR-Cas9 system specifically knocks out the Cdk5 gene, leading to a significant downregulation of PD-L1 expression on the surface of tumor cells, blocking the transmission of immune checkpoint inhibitory signals to the PD-1 receptors on T cells. This mechanism triggers a robust T cell-mediated immune response, achieving highly efficient killing of tumor cells. Reproduced with permission [45]. Copyright 2020 Elsevier by CC-BY.

PD-L1 protein levels are finely tuned by various upstream regulators that control its trafficking and degradation. For instance, CMTM6 and CDK5 have been identified as key chaperones that prevent PD-L1 from entering the lysosomal degradation pathway, thereby maintaining its presence on the cell membrane. We utilized a biodegradable cationic polymer, poly(β-amino ester) (PBAE), to deliver a CRISPR-Cas9 plasmid targeting the Cdk5 gene (Figure 6) [45]. By knocking out Cdk5, they interfered with the IRF2/IRF2BP2 pathway, leading to the downregulation of PD-L1 in tumor cells. This strategy effectively remodeled the immunosuppressive microenvironment and enhanced T cell infiltration and antitumor immune responses in both melanoma and triple-negative breast cancer models, providing a preclinical foundation for CRISPR-based genome editing to downregulate PD-L1 in cancer therapy. Burks et al. found that in pancreatic ductal adenocarcinoma (PDAC), the high expression of the interferon-stimulated gene 15 (ISG15) pathway—induced by type I interferons (IFN-α/β)—is closely associated with the KRAS-driven malignant phenotype and PD-L1 upregulation [46]. CRISPR-mediated knockdown of ISG15 inhibited tumor proliferation, downregulated PD-L1 expression, promoted CD8^+^ T cell infiltration, and reduced regulatory T cells (Tregs), thereby delaying tumor growth. The synergy between ISG15 knockdown and anti-PD-1 therapy significantly enhanced antitumor immunity, suggesting that targeting ISG15 is an effective combination strategy for PDAC immunotherapy. To targeting knocking out the CTNNB1 gene (encoding β-catenin), thereby inhibiting the Wnt/β-catenin signaling pathway to downregulates PD-L1 expression, He et al. constructed a multifunctional delivery system based on natural polymers, functionalized with aptamer AS1411-modified hyaluronic acid (AHA) and nuclear-targeting peptide TAT-NLS-modified hyaluronic acid (PHA), to deliver CRISPR-Cas9 plasmids for CTNNB1 [47]. This carrier specifically targets the tumor cell nucleus to achieve high gene-editing efficiency thereby reversing tumor immunosuppression and restoring T cell antitumor activity.

The dysregulated metabolism of tumor cells, particularly the “Warburg effect” characterized by high glycolysis and subsequent lactate accumulation, is a primary driver of PD-L1 upregulation and T cell exhaustion. This lactate not only acidifies the tumor milieu to suppress effector cells, but also drives histone lactylation to stabilize HIF-1α and PD-L1. Reinforced by a PKM2/HIF-1α positive feedback loop, this metabolic-epigenetic coupling locks chromatin accessibility into a tolerogenic state. It skews inflammatory signaling to favor IL-10 over pro-inflammatory factors (IFN-γ, TNF-α, and IL-12), ultimately blunting dendritic cell maturation, inducing M2 macrophage polarization, enriching regulatory T cells, and exhausting cytotoxic T lymphocytes. We reported a pioneering metabolic-immune dual reprogramming strategy using multifunctional nanoparticles (PPPt^IV^ NPs) for the co-delivery of a cisplatin prodrug (Pt IV) and CRISPR-Cas9 plasmids targeting PKM2 to treat head and neck squamous cell carcinoma (HNSCC) [91]. This platform leverages cisplatin to ICD while simultaneously silencing PD-L1. Crucially, targeted PKM2 knockdown dismantles the epigenetic–metabolic axis system and addresses the aberrant lactate metabolism within the HNSCC microenvironment. By correcting metabolic-immune crosstalk, the PPPt^IV^ NPs reverse lactate-mediated immunosuppression, downregulates HIF-1α/PD-L1 and reshapes the cytokine profile, thereby revitalizing CD8^+^ T cell activity and demonstrating superior synergistic therapeutic outcomes in platinum-resistant models.

KAT8 is identified as a critical nucleator for LLPS-mediated transcriptional condensates, which are essential transcription factors to drive persistent PD-L1 expression [35]. In hepatocellular carcinoma, KAT8-mediated acetylation of IRF1 at lysine 78 alleviates electrostatic repulsion within its DNA-binding domain, triggering multivalent interactions that drive LLPS into nuclear transcriptional condensates. By altering chromatin accessibility via H4K16ac and uninterruptedly recruiting the transcriptional machinery, these condensates convert a transient interferon response into chronic, unremitting PD-L1 transcription. This persistent axis constantly replenishes surface PD-L1, reinforces immune checkpoint density, and ultimately perpetuates T cell exhaustion within the tumor microenvironment. Utilizing a KAT8 siRNA can disrupt these biophysical condensates at the source. By preventing the formation of transcriptional hubs, this strategy achieves deep and durable PD-L1 blockade and promotes M1-type macrophage polarization, offering a revolutionary perspective on overcoming immune resistance by targeting the biophysical properties of the TME.

Consequently, intervening in upstream regulatory nodes of PD-L1 using gene-editing tools can systemically inhibit its expression at the transcriptional level. However, it is crucial to recognize that these upstream regulatory nodes—such as CTNNB1, CDK5, ISG15, and KAT8—are highly pleiotropic. They govern not only PD-L1 expression but also essential physiological processes, including tissue regeneration, neuronal function, antiviral immunity, and the homeostatic polarization of normal macrophages. Consequently, systemic or non-selective intervention against these targets risks severe unintended effects, such as gastrointestinal toxicity, immunosuppression, or neurotoxicity. This underscores the critical necessity of employing advanced, tumor-targeted delivery platforms. By utilizing responsive nanomaterials and functionalized polymeric carriers (e.g., PBAE or targeted hyaluronic acid systems), gene-editing tools can be confined to the tumor microenvironment. This spatially restricted delivery strategy is vital for mitigating off-target systemic toxicities while maximizing therapeutic efficacy, particularly for challenging indications like orthotopic pancreatic tumors, paving the way for effective clinical translation. This approach acts synergistically with existing immune checkpoint blockade (ICB) therapies and effectively overcomes adaptive resistance in tumors.

### 3.4. Enhancing CAR-T Cell Therapy via Gene Editing-Mediated Regulation of PD-1

In the field of CAR-T cell therapy, modulating PD-1/PD-L1 signaling through gene editing has become a critical direction for enhancing therapeutic efficacy, minimizing toxic side effects, and developing universal products. Current research primarily focuses on the construction of low-immunogenicity universal CAR-T cells, the optimization of gene editing and integration strategies, and the localized delivery of immunomodulatory molecules. Regarding the construction of low-immunogenicity universal CAR-T (UCAR-T) cells, gene editing is employed to mitigate allogeneic rejection and PD-1-mediated self-inhibition. Ren et al. utilized a multiplex gene-editing strategy to simultaneously knock out TCR, B2M, and PD1 genes, developing UCAR-T cells with both reduced immunogenicity and enhanced antitumor activity [92]. Their study demonstrated that triple-knockout CAR-T cells significantly decreased alloreactivity while maintaining potent cytotoxicity. Furthermore, the manufacturing process can be integrated into GMP-standard workflows, providing a feasible path for “off-the-shelf” immune cell products. Similarly, Choi et al. used CRISPR-Cas9 to concurrently knock out the T cell receptor (TRAC), β2-microglobulin (B2M), and PD-1 (PDCD1) to construct low-immunogenicity universal EGFRvIII CAR-T cells [93]. In glioblastoma models, this strategy effectively overcame immunosuppression driven by therapy-induced PD-L1 upregulation, significantly enhancing antitumor potency. In a Phase I clinical trial targeting mesothelin-positive solid tumors, Wang et al. utilized CRISPR-Cas9 to construct PD-1/TCR double-knockout CAR-T cells (MPTK-CAR-T) [94]. While the study confirmed preliminary safety, it was observed that the loss of endogenous TCR might compromise the persistence of CAR-T cells, suggesting that TCR signaling plays a vital role in cell survival within the solid tumor microenvironment.

In terms of optimizing gene editing and integration strategies, research focuses on improving editing precision, safety, and product uniformity. Zhang et al. utilized CRISPR-Cas9 to integrate an anti-CD19 CAR sequence into the AAVS1 safe harbor locus while simultaneously knocking out the PD-1 gene, creating a non-viral, bifunctionally optimized CAR-T cell [95]. This strategy combines the safety of non-viral preparation with the precision of site-specific integration. Preclinical and clinical results confirmed its sustained and efficient antitumor activity while simplifying the manufacturing process and reducing costs. Hu et al. developed a CRISPR/Cas9-based non-viral site-specific integration CAR-T product (PD1-19bbz) by integrating the anti-CD19 CAR sequence specifically into the PD1 locus for treating relapsed/refractory B-cell non-Hodgkin lymphoma [96]. Phase I clinical trial results showed that all 21 enrolled patients achieved clinical responses, with 18 (85.7%) obtaining complete remission and a median progression-free survival of 19.5 months, providing a significant foundation for the clinical translation of non-viral CAR-T products.

**Figure 7:**
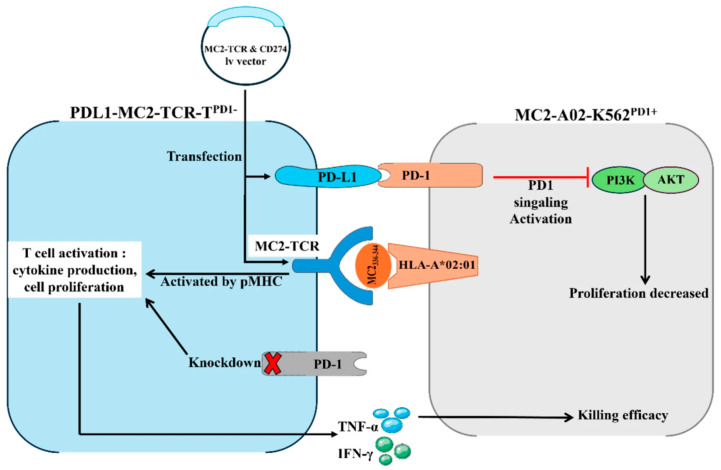
Interactions between engineered T cells and target cancer cells. The PDL1-MC2-TCR-TPD1-cells are engineered to co-express a MAGE-C2-specific recombinant TCR and PD-L1 proteins in normal human CD8^+^ T cells, with the PD-1-encoding PDCD1 gene knocked out. The exogenously expressed PD-L1 activates the PD-1 receptor on the surface of cancer cells while maintaining PD-1 suppression in the T cells. Activation of PD-1 inhibits PI3K-mediated carcinogenic signaling and subsequent cell proliferation. Binding of MC2-TCR with MC2 pMHC induces T cell activation and directs cytotoxicity toward target cells through the secretion of cytokines TNF-α and IFN-γ. Silencing of PD-1 in T cells enhances T cell activation and expansion. Red lines denote inhibitory effects, and “×” indicates knockdown of PDCD1 genes. Reproduced with permission [98]. Copyright 2025 Springer Nature by CC-BY.

For the localized delivery of immunomodulatory molecules, engineering CAR-T cells to secrete immune checkpoint inhibitors can enhance local immune responses while reducing systemic toxicity. Rafiq et al. modified CAR-T cells to secrete a PD-1-blocking single-chain variable fragment (scFv) [97]. This scFv enhances the antitumor activity of both CAR-T cells and bystander tumor-specific T cells via paracrine and autocrine mechanisms. In mouse models of hematologic and solid tumors with high PD-L1 expression, its efficacy was comparable to or superior to systemic checkpoint blockade, while potentially avoiding the risks of systemic toxicity. Furthermore, Zhao et al. expanded the dimensions of engineered T cells by using CRISPR/Cas9 to knock out the PDCD1 gene in TCR-T cells and co-expressing a MAGE-C2-specific TCR and PD-L1 (Figure 7) [98]. This PDL1-MC2-TCR-TPD1-cell achieves “dual regulation”: (1) PD-L1 selectively activates PD-1 on tumor cells to inhibit the PI3K-AKT-mTOR oncogenic axis; (2) the TCR recognizes the MC2-pMHC complex to exert targeted cytotoxicity; and (3) the loss of endogenous PD-1 in T cells relieves self-inhibition, enhancing activation and expansion. Compared to conventional ICB, this strategy avoids non-targeted effects on bystander tissues and prevents complications such as “cytokine storms,” providing a novel adoptive immunotherapy strategy for T cell malignancies.

In summary, modulating PD-1/PD-L1 signaling via gene editing significantly enhances the efficacy and safety of CAR-T cell therapy and drives the development of universal products. Future research should focus on optimizing editing specificity, balancing cell persistence with functional maintenance, and accelerating the clinical validation of engineered cell products.

## 4. Clinical Challenges of Gene Medicines Targeting PD-1/PD-L1

While gene therapies targeting the PD-1/PD-L1 axis have demonstrated immense potential in preclinical models, their clinical translation is still hindered by critical technological bottlenecks, safety concerns, and ethical challenges.

First, delivery efficiency and specificity remain the primary prerequisites. Current strategies are bifurcated into ex vivo modification (e.g., CAR-T cells) and in vivo regional/systemic editing. Although ex vivo approaches offer high controllability, they are plagued by manufacturing complexity, exorbitant costs, and limited scalability. Conversely, in vivo editing demands extreme precision and stability from delivery systems. A persistent hurdle is achieving tumor-specific accumulation while avoiding non-specific sequestration by the liver and other reticuloendothelial organs. Recent advancements, such as the high-throughput molecular barcoding, have begun to address this by identifying hybrid LNPs with organ-specific tropism (e.g., lung-targeted delivery), yet achieving uniform editing efficiency within the heterogeneous and dense stroma of solid tumors remains a formidable barrier. To overcome these physical barriers, particularly in highly desmoplastic malignancies such as pancreatic cancer, researchers are actively developing dynamically reconfigurable biomaterials. For instance, stimuli-responsive systems utilizing peptide self-assembly can undergo structural transformations (e.g., from spherical micelles to nanofibers) upon entering the TME. This adaptive morphology allows the nanocarriers to penetrate deep into the dense extracellular matrix and efficiently deliver gene-editing payloads [99]. Simultaneously, to address the pressing issues of systemic off-target effects and in vivo instability, emerging surface-engineering strategies are being rapidly adopted. A notable advancement is the application of in situ biomineralization on viral vectors or polymeric nanocarriers. By enveloping the gene-delivery vehicle in a responsive mineralized shell (such as calcium phosphate or manganese dioxide), the CRISPR/Cas machinery is physically shielded from enzymatic degradation and immune clearance during systemic circulation. This mineral coating is specifically designed to undergo rapid dissolution only within the acidic and redox-active TME, thereby ensuring highly localized genome editing and drastically minimizing off-target toxicity in healthy organs [100]. Furthermore, the efficiency of large-fragment “knock-in” (e.g., site-specific CAR integration) continues to lag far behind simple gene knockouts, representing a key technical bottleneck in process optimization.

Second, safety risks—particularly off-target effects—are an unavoidable concern in genome editing. Unintended mutations or structural variations, such as chromosomal translocations and large-scale deletions during double-strand break (DSB) repair, necessitate rigorous long-term genomic assessment. Beyond genomic stability, the biological response of edited cells warrants close monitoring. Engineered immune cells may trigger severe adverse events like cytokine release syndrome (CRS) or neurotoxicity due to over-activation. Simultaneously, for edited somatic cells (e.g., tumor cells), the risks of long-term survival, compensatory pathway activation, or even secondary malignant transformation must be evaluated. Moreover, the immunogenicity of exogenous components (such as Cas9 proteins or viral vectors) can elicit host immune responses, leading to the rapid clearance of therapeutic cells and diminishing long-term efficacy. It’s worth noting that significant progress has been achieved in viral vector delivery through capsid engineering and synthetic promoter design, effectively enhancing tissue targeting and mitigating off-target risks. Capsid engineering, utilizing methods such as directed evolution and rational design, has generated novel virus (AAV) variants with modified tropisms. For instance, the engineered variant AAV-PHP.eB displays dramatically improved blood–brain barrier (BBB) penetration compared to native AAV9, enabling robust gene delivery to the central nervous system via systemic administration [101]. To further restrict transgene expression to target organs and reduce systemic toxicities, tissue-specific promoters are increasingly utilized in clinical-stage pipelines, such as Hemgenix and Zolgensma, which have been approved by FDA. This dual-control strategy—combining capsid tropism with transcriptional regulation—represents a critical paradigm for the clinical translation of effective viral gene therapies [102].

An important lesson from the ICB experience is that PD-L1 expression alone is an imperfect biomarker. Deep learning models that integrate multi-omics data including tumor mutational burden, neoantigen load, immune cell deconvolution, and radiomic features consistently outperform PD-L1 IHC in predicting immunotherapy response. For PD-L1 gene therapy, this implies that patient selection cannot rely solely on PD-L1 expression levels. Instead, pre-treatment assessment of the tumor immune ecosystem including the presence of tumor-infiltrating lymphocytes, the activation state of antigen-presenting cells, and the abundance of immunosuppressive populations (Tregs, MDSCs) may be equally critical. Systems-biology approaches that integrate these multi-dimensional features into predictive models will be essential for identifying patients most likely to benefit from PD-L1 gene editing [103].

Beyond hypothesis-driven studies, unbiased functional genomics approaches have begun to map the PD-L1 regulatory network at scale. Genome-wide CRISPR screens in lung cancer cells not only recovered canonical regulators (IFNGR1, JAK2, IRF1) but also identified unexpected nodes in one-carbon metabolism including GART, SHMT2, and MTHFD2 as essential for PD-L1 expression. Mechanistically, GART knockout reduces PD-L1 by impairing purine synthesis and dampening JAK-STAT signaling. This finding expands the metabolic-immune crosstalk axis beyond lactate and glycolysis to include folate-dependent one-carbon metabolism, and suggests that PD-L1 regulation is integrated with core cellular biosynthetic pathways. Importantly, such screens also reveal redundancy: multiple metabolic nodes converge on the same JAK-STAT output, implying that targeting any single node may be circumvented [104].

Tumor heterogeneity represents a fundamental challenge for PD-L1-targeted gene therapy. Even if a delivery system achieves 90% PD-L1 knockout at the bulk level, the remaining 10% of cells potentially those in hypoxic niches with limited perfusion or those with distinct genetic subclones may serve as a reservoir for relapse. Moreover, spatial heterogeneity in the TIME means that PD-L1 expression is often highest at the invasive margin where T cells are present (adaptive resistance) and lower in the tumor core (innate resistance). A CRISPR-based approach that eliminates PD-L1 uniformly across all tumor cells could theoretically overcome this, but current delivery technologies rarely achieve homogeneous intratumoral distribution. Multi-omics analyses of tumor heterogeneity further reveal that PD-L1 expression is embedded within broader transcriptional programs; thus, PD-L1 knockout may be compensated by upregulation of alternative checkpoints (TIM-3, LAG-3, TIGIT) in surviving subclones, a phenomenon that requires longitudinal monitoring and potentially combinatorial gene editing [105].

Finally, the path to clinical translation requires the establishment of standardized Chemistry, Manufacturing, and Controls (CMC) and robust Quality Control (QC) systems to ensure batch-to-batch consistency. Especially for multifunctional nanoplatforms, the complexity here makes it really hard to be translated into GMP-compatible clinical products. Clinical trial designs must transition toward precision medicine, identifying optimal patient cohorts and determining the most effective dosing regimens and delivery routes. From an ethical and regulatory standpoint, while somatic editing does not involve germline inheritance, the permanence of genetic changes and the equity of access to these high-cost therapies necessitate a delicate balance between scientific innovation and patient benefit.

## 5. Conclusions and Future Perspectives

Gene therapy provides a highly controllable platform for modulating the PD-1/PD-L1 pathway, offering a mechanistic advantage over traditional antibody-based immune checkpoint blockade (ICB) by neutralizing inhibitory signaling directly at its source. Some gene-based targeted therapies have been under clinical trial (e.g., NCT02793856, NCT03545815 and NCT04213469). While strategies such as CRISPR/Cas-mediated disruption and RNA interference have demonstrated significant potential in reversing adaptive immune resistance, their clinical translation remains constrained by delivery inefficiencies, dense stromal barriers, and the risks of off-target toxicity.

Moving forward, overcoming these translational bottlenecks will require a paradigm shift toward multidisciplinary and forward-looking strategies. The next generation of gene medicines in this field is expected to evolve along the following emerging trajectories:

- Advanced Polymeric and Biomimetic Nanostructures: To breach the formidable physical barriers of “cold” and highly desmoplastic tumors (e.g., pancreatic cancer), dynamic delivery vehicles are critically needed. Emerging technologies, such as responsive polymer materials and peptide self-assembly systems, offer transformative potential. These biomimetic structures can undergo morphology transformations in vivo, enabling deep tissue penetration and controlled cargo release within the TME.
- In Situ Mineralization for Unprecedented Stability: The delicate nature of gene editors (like Cas RNPs) demands robust protection during systemic circulation. Future research should heavily leverage in situ biomineralization strategies to encapsulate gene-delivery vehicles within an inorganic, protective shell. This mineralized armor not only shields the payload from enzymatic degradation but also intrinsically dissolves in the acidic TME, ensuring highly targeted, stimuli-responsive release and effectively eradicating systemic off-target risks.
- Next-Generation, DSB-Free Genome Editing: The field must rapidly transition from conventional CRISPR/Cas9 tools that generate DNA double-strand breaks (DSBs) to advanced technologies like base editing (BE) and prime editing (PE). These sophisticated tools will enable multiplexed, controllable genomic alterations (e.g., engineering universal CAR-T cells) without the perilous risks of chromosomal translocations or large-scale genomic instability.
- Spatiotemporal Multi-dimensional Synergy: Ultimately, genetic interruption of the PD-1/PD-L1 axis will not stand alone. Future therapies will intricately design intelligent nanoplatforms to co-deliver genetic modulators alongside metabolic reprogrammers or innate immune agonists, orchestrating a comprehensive, spatiotemporally synchronized assault on tumor heterogeneity.

Beyond individual regulators, emerging evidence indicates that oncogenic signaling pathways including Wnt/β-catenin, Notch, JAK/STAT, p53, and PTEN operate as integrated networks that collectively shape PD-L1 expression and immune evasion. These pathways exhibit both independent and cross-regulatory interactions, suggesting that optimal PD-L1 suppression may require simultaneous targeting of multiple nodes rather than any single pathway [106,107]. The cGAS-STING pathway exemplifies the complexity of pathway crosstalk in PD-L1 regulation. While STING activation can enhance antitumor immunity by promoting type I interferon responses, sustained or cell-type-specific STING signaling may paradoxically upregulate PD-L1 via STAT1/IRF1 activation. This dual functionality context-dependent and cell-type-specific underscores the importance of precise, temporally controlled pathway modulation rather than indiscriminate activation [108]. Beyond PD-1/PD-L1, systematic pan-cancer analyses have identified additional immune regulatory molecules with therapeutic potential. For instance, DKK3 functions as an independent inhibitor of CD8^+^ T cell activation, and combined DKK3/PD-1 blockade produces synergistic antitumor effects [109]. Similarly, FCER1G has been identified as a common regulator of anti-tumor immune responses across tumor stages [110]. These findings suggest that future gene therapy strategies may need to simultaneously target multiple immune checkpoints or regulatory molecules to overcome redundancy in tumor immune evasion networks.

Not all PD-L1 regulatory mechanisms are equal. Core genetic drivers (e.g., PTEN loss, RAS activation) establish a baseline of PD-L1 expression that renders tumors ‘pre-primed’ for immune evasion. TME modulators such as IFN-γ and lactate act on this baseline, amplifying PD-L1 in a context-dependent manner. Critically, adaptive resistance, the upregulation of PD-L1 in response to therapy, often re-uses the same modulatory pathways (e.g., IFN-γ–STAT1 signaling) but is distinguished by its temporal emergence under selective pressure. Post-translational stabilizers (CMTM6, CDK5) operate independently of transcriptional control, providing a parallel layer of regulation that can maintain PD-L1 even when transcription is blocked.

Moreover, redundancy is evident among certain regulators. For example, both CMTM6 and CMTM4 can protect PD-L1 from lysosomal degradation, and dual targeting may be required for full effect. Similarly, multiple cytokines (IFN-γ, TNF-α, IL-6) converge on STAT and NF-κB pathways. Conversely, synergy occurs when targeting orthogonal layers: combining HIF-1α knockout (reducing transcriptional induction under hypoxia) with Cdk5 knockout (destabilizing PD-L1 protein) may produce greater-than-additive effects.

In conclusion, by merging cutting-edge materials science with precision genome editing, PD-1/PD-L1-targeted gene therapies will transcend current limitations, charting a definitive course toward curative and personalized cancer immunotherapies.

## Figures and Tables

**Table 1:** Biological intervention point of PD-1/PD-L1 blockade.

Mechanistic Level	Gene Tool	Biological Advantage	Limitation	Ref
Genomic DNA	CRISPR/Cas9	Permanent knockout;eliminates nuclear PD-L1 function	Off-target risk; DSB toxicity	[27,28,29,30,31,32]
mRNA	siRNA/shRNA	Transient, reversible	Requires continuous delivery, RISC saturation risk	[33,34,35,36,37,38,39,40,41,42,43,44]
Transcriptional regulation	CRISPRi, targeting HIF-1α/β-catenin	Modulates upstream drivers	Indirect; may affect other genes	[45,46,47]
Post-translational (stability)	Targeting CMTM6/ CDK5	Preserves membrane-proximal regulation	Chaperone redundancy	[45,48,49]
Biophysical (LLPS)	KAT8 siRNA	Disrupts transcriptional condensates	Novel; long-term effects unknown	[35]

## Data Availability

No new data were created or analyzed in this study. Data sharing is not applicable to this article.
